# Persistent CD8^+^ T cell proliferation and activation in COVID-19 adult survivors with post-acute sequelae: a longitudinal, observational cohort study of persistent symptoms and T cell markers

**DOI:** 10.3389/fimmu.2023.1303971

**Published:** 2024-01-23

**Authors:** Stephanie M. LaVergne, Taru S. Dutt, Kim McFann, Bridget A. Baxter, Tracy L. Webb, Kailey Berry, Maddy Tipton, Sophia Stromberg, Brian M. Sullivan, Julie Dunn, Marcela Henao-Tamayo, Elizabeth P. Ryan

**Affiliations:** ^1^ Department of Environmental Radiological and Health Sciences, Colorado State University, Fort Collins, CO, United States; ^2^ Department of Microbiology, Immunology and Pathology, Colorado State University, Fort Collins, CO, United States; ^3^ University of Colorado Health, Medical Center of the Rockies, Loveland, CO, United States; ^4^ Department of Clinical Sciences, Colorado State University, Fort Collins, CO, United States; ^5^ Department of Molecular, Cellular, and Integrative Neurosciences, Colorado State University, Fort Collins, CO, United States; ^6^ Department of Food Science and Human Nutrition, Colorado State University, Fort Collins, CO, United States; ^7^ Department of Immunology and Microbiology, Scripps Research, La Jolla, CA, United States

**Keywords:** post-acute sequelae of COVID-19, post-COVID, COVID-19, CD8+ T cell, cytotoxic T cells, SARS-CoV-2

## Abstract

**Introduction:**

Post-acute sequelae of COVID-19 affects the quality of life of many COVID-19 survivors, yet the etiology of post-acute sequelae of COVID-19 remains unknown. We aimed to determine if persistent inflammation and ongoing T-cell activation during convalescence were a contributing factor to the pathogenesis of post-acute sequelae of COVID-19.

**Methods:**

We evaluated 67 individuals diagnosed with COVID-19 by nasopharyngeal polymerase chain reaction for persistent symptoms during convalescence at separate time points occurring up to 180 days post-diagnosis. Fifty-two of these individuals were evaluated longitudinally. We obtained whole blood samples at each study visit, isolated peripheral blood mononuclear cells, and stained for multiple T cell activation markers for flow cytometry analysis. The activation states of participants’ CD4^+^ and CD8^+^ T-cells were next analyzed for each of the persistent symptoms.

**Results:**

Overall, we found that participants with persistent symptoms had significantly higher levels of inflammation at multiple time points during convalescence when compared to those who fully recovered from COVID-19. Participants with persistent dyspnea, forgetfulness, confusion, and chest pain had significantly higher levels of proliferating effector T-cells (CD8^+^Ki67^+^), and those with chest pain, joint pain, difficulty concentrating, and forgetfulness had higher levels of regulatory T-cells (CD4^+^CD25^+^). Additionally, those with dyspnea had significantly higher levels of CD8^+^CD38^+^, CD8^+^ Granzyme B^+^, and CD8^+^IL10^+^ cells. A retrospective comparison of acute phase inflammatory markers in adults with and without post-acute sequelae of COVID-19 showed that CD8^+^Ki67^+^ cells were significantly higher at the time of acute illness (up to 14 days post-diagnosis) in those who developed persistent dyspnea.

**Discussion:**

These findings suggest continued CD8+ T-cell activation following SARS-CoV-2 infection in adults experiencing post-acute sequelae of COVID-19 and that the increase in T regulatory cells for a subset of these patients represents the ongoing attempt by the host to reduce inflammation.

## 1 Introduction

Severe acute respiratory syndrome coronavirus 2 (SARS-CoV-2) has taken the lives of millions worldwide but could leave an even greater number of survivors with debilitating sequelae (1). Many COVID-19 survivors experience persistent symptoms for months after the virus is no longer detectable by nasopharyngeal polymerase chain reaction (PCR) (2–5). A variety of persistent symptoms and manifestations that are described by patients are classified as post-acute sequelae of COVID-19 (PASC) (4, 6–8). Precise definitions of PASC are lacking, and therefore studies evaluating this syndrome remain challenging (9, 10). Importantly, mild disease and asymptomatic cases may go on to develop persistent symptoms or PASC, including younger individuals (11, 12). Critical gaps in knowledge exist on the pathophysiology of PASC and longitudinal analysis of patients merits attention given the adverse social, economic and health impacts with decreased quality of life (13–16).

Understanding PASC is crucial to disease control and management. Post-viral syndromes have been well described in association with other viral illnesses, and persistent immune dysfunction may play a vital role in these conditions and in precipitating autoimmune disease (17–22). It is possible that tissue damage from SARS-CoV-2 and the immune response it elicits may lead to new disease or amplification of undiagnosed underlying medical conditions that contribute to the development of PASC. Elevated inflammatory markers including D-dimer, IL-6, IFN-β/PXT3/IFN-γ/IFN-lambda/IL-6 combination, CD56^+^ NK cells, granulocytes, neutrophils, CXCR3^+^ monocytes, and C-reactive protein (CRP) have been found in PASC patients during convalescence (23–27). Liao et al. found that elevated IL-6 levels were associated with persistent pulmonary lesions on CT scans (23). Additionally, Raman et al. found that elevated CRP correlated with extra-pulmonary MRI abnormalities in PASC patients 2-3 months after disease onset (28). Lower frequencies of the degranulation marker CD107a in CD8^+^T cells have been observed in PASC patients four months after initial infection (29), suggestive of cytotoxic T cell (CTL) death during degranulation. Persistent inflammation may cause significant pathophysiology and lingering symptoms and provided rationale for determining if patients with PASC had elevated cellular markers of inflammation in the weeks to months following initial infection. The objective of this study was to determine if persistent inflammation using a suite of T cell activation markers in peripheral blood are contributing factors involved in PASC.

## 2 Materials and methods

### 2.1 Participant enrollment

We aimed to evaluate ongoing T cell activation and persistent inflammation in COVID-19 survivors as contributing factors for PASC. We conducted a longitudinal observational cohort study with evaluations at 4 separate time points for biospecimen and clinical symptom data collection used in this analysis. This study is comprised of sixty-seven acute and convalescent adult participants diagnosed with COVID-19 that enrolled in the Northern Colorado SARS-CoV-2 Biorepository (NoCo-COBIO) that was established at Colorado State University (CSU) as part of a community-based collaboration with the University of Colorado Health (UCH) in Fort Collins, CO (30). Inclusion criteria for all participants included (1) a positive SARS-Co-V-2 polymerase chain reaction (PCR) test, (2) at least 18 years of age, and (3) not currently pregnant or incarcerated. The recruitment of participants took place through UCH Northern Colorado hospitals, including Poudre Valley Hospital (PVH) in Fort Collins, Medical Center of the Rockies (MCR) in Loveland, and Greeley Hospital in Greeley, using emails, recruitment flyers, and health department screening. Participants were either enrolled from the community or from the inpatient hospital setting (PVH, MCR or Greeley Hospitals). Participants consented to undergo at least 3 study clinic visits: at time of enrollment, approximately one month after enrollment, 4 months after enrollment, and 6 months after enrollment if a fourth visit was consented and completed. Written informed consent was obtained from all participants prior to enrollment. Complete account of recruitment, enrollment, and data acquisition with rationale has previously been described (30).

In total, 116 participants were enrolled in the biobank, however participants had to complete at least one convalescent visit after their acute illness to be eligible for data analysis for this manuscript to determine if lingering symptoms were present. Of the 116 participants, many did not follow up for a convalescent visit after the acute phase of illness, some participants died, and some participants had inadequate specimen quantity to perform Peripheral blood mononuclear cells (PBMC) analysis. Therefore, 67 participants were used for this data analysis. Participants were enrolled at different timepoints following a COVID-19 PCR positive test result. Some participants were enrolled during the acute phase of their disease (within 14 days of a positive PCR test) (N=24), and some were enrolled during convalescence (N=43).

These 67 participants’ SARS-CoV-2 PCR positive test dates ranged from March 17, 2020, through February 26, 2021. All included participants completed their study visits between July 8, 2020, and April 7, 2021. Fifty-two adults had two or more visits. At the time of enrollment, disease severity was determined for each participant based on the Yale Impact Score: mild (no oxygen required), moderate (1-5L of oxygen required), and severe (>5L of oxygen required).

This biorepository and written informed consent were approved by CSU’s Research Integrity and Compliance Review Board (IRB protocol ID 2105 and 20-10063H), and the UCH IRB (Colorado Multiple IRB 20–6043), and is registered at ClinicalTrials.gov (NCT04603677). This biorepository was in accordance with the 1964 Helsinki Declaration and its 2013 amendments.

### 2.2 Peripheral blood mononuclear cell isolation

Each study participant provided approximately 40mL of whole blood in five 8mL cell preparation tubes (CPTs). Peripheral blood mononuclear cells (PBMCs) were isolated using the methodology previously outlined (31). Briefly, CPT tubes were centrifuged at 1500g for 30 minutes with the brake off. The buffy coat was aspirated from each CPT, transferred to a 50mL conical tube, and washed twice with phosphate buffer saline (PBS). The pellet was resuspended in 5mL PBS, the cells were counted, and 1x10 (6) cells were added to a V-bottom 96-well plate in triplicate for flow cytometric staining.

### 2.3 Peripheral blood mononuclear cell staining, flow cytometry, and data extraction

Peripheral blood mononuclear cells were incubated with 100µL of 1x Brefeldin A solution (5 mg/ml) (Fischer Scientific, Pittsburg, PA) at 37°C with 5% CO_2_. After two hours, cells were washed with 150µL FACS buffer (PBS, 2% heat-inactivated fetal bovine serum, and 0·05 percent sodium azide) and Fc receptors were blocked using 1:200 dilution of human Fc block (BD Biosciences). Cells were incubated for 15 minutes at 4°C and further stained with 50µL of surface antibody cocktail and incubated for 30 minutes at 4°C in the dark. Following surface labeling, cells were washed and fixed for one hour at room temperature using 100µL 1x fix/perm staining buffer (eBiosciences). Cells were washed with 1x permeabilization buffer (eBiosciences) and incubated overnight at 4°C in the dark with an intracellular antibody cocktail. The following day, the cells were washed with 1x permeabilization buffer, resuspended in 200µL FACS buffer and acquired using a Cytek four-laser Aurora spectral flow cytometer. 50,000 events in the leukocyte gate were recorded.

Data analysis was performed using FlowJo (BD Biosciences) software version 10.8.1. Cells were gated on live lymphocytes, single cells, CD3^+^ cells, and either CD8^+^ or CD4^+^ cells. CD4^+^ cells were evaluated for CD25^+^, HLADR^+^, IFNγ^+^, IL-10^+^, and TNFα^+^. CD8^+^ cells were evaluated for CD38^+^, PD1^+^, Granzyme-B^+^, IFNγ^+^, TNFα^+^, IFNγ^+^TNFα^+^, IL-10^+^, and Ki67^+^. Data were extracted as the frequency of the parent population (FOP) or frequency of the total lymphocyte population (FOL) for each sample.

### 2.4 Enzyme-linked immunosorbent assay for anti-receptor binding domain IgG detection

Enzyme-linked immunosorbent assay (ELISA) was used to test antibody binding to SARS-CoV-2 receptor binding domain (RBD) (catalog numbers 40592-V08H, Sino Biological US Inc., Wayne, PA, USA) (31, 32). High-binding 96-half-well microplates (Corning Life Sciences, Tewksbury, MA, USA) were coated with 50ng RBD protein in PBS overnight at 4°C. The next day, plates were washed five times with PBS + 0.5% Tween 20 and treated with blocking buffer (PBS + 0.5% Tween 20 + 2% BSA + 2% normal goat serum [Jackson ImmunoResearch Inc., West Grove, PA, USA]) for two hours. Different dilutions of human plasma in blocking buffer were added and incubated for one hour. Plates were washed and incubated for one hour with HRP-conjugated anti-human IgG antibody (Jackson ImmunoResearch Inc.) (1:10,000 dilution). The process was stopped by adding 50µL of 1N sulfuric acid to 100L TMB substrate (Thermo Fisher Scientific, Rockford, IL, USA). BioTek Synergy 2 plate reader was used to measure OD at 450 nm (BioTek Instruments Inc., Winooski, VT, USA).

### 2.5 Post-acute sequelae of COVID-19 (PASC) symptom evaluation

At each convalescent visit, participants were evaluated for persistent symptoms by completing a questionnaire that screened for 70 different persistent symptoms, detailed in our prior report (30). We defined PASC herein as participants experiencing at least 1 major symptom (dyspnea, fatigue, confusion, difficulty concentrating, forgetfulness, joint pain or chest pain) or 3 minor symptoms (from symptom list reported on the questionnaire) that remain present 14 days after the diagnostic SARS-CoV-2 PCR positivity. The symptoms must be reported as new symptoms and not present prior to COVID-19.

### 2.6 Statistical analysis

Statistical analysis was completed for each of the inflammatory markers, comparing those with and without PASC broadly, and for those specifically with or without dyspnea, fatigue, confusion, difficulty concentrating, forgetfulness, joint pain, and chest pain. When we didn’t find statistical differences between those with and without PASC, we analyzed by individual symptom. PASC is not homogenous and various symptoms could represent distinct pathophysiology, and statistical comparisons of inflammatory markers by the presence of individual symptoms merits attention. We completed statistical analysis on the most common PASC symptoms in our cohort.

Each cellular marker or intracellular cytokine was measured as a percent of the parent cell population (CD8^+^ or CD4^+^), as well as the percent of total lymphocytes in the sample (n=67). The flow cytometric data were evaluated for the distributional assumption of normality, and due to highly left-skewed curves for percent change, non-parametric methods were used. The Wilcoxon Sign-Rank Test was applied to investigate differences between two groups involving PASC and PASC symptoms. A power calculation for estimating sample size needed to assess relationships between T cell activation markers and PASC was not completed for this longitudinal, observational cohort investigation. This study was not able to predict the number of individuals that would clinically present with PASC and was not powered accordingly. Data were reported as Median and Interquartile Range. P < 0.05 was applied for statistical significance without adjustment for multiple tests. Of the 67 participants in this study, 52 adults had more than two blood collections with flow cytometric analysis of PBMCs between 1-6 months after the PCR+ test for COVID-19. The individual components of PASC including cognitive disorders, fatigue, chest pain, joint pain, and difficulty breathing were examined using mixed model longitudinal data analyses with an auto regressive error structure. Statistical significance was calculated using ANOVA with Tukey HSD. A P value of <0.05 was considered significant. When comparing two groups, for example, when comparing inflammatory markers in patients with and without symptoms, the Wilcoxon signed-rank test was used. Data is reported in the results section as Median in parenthesis, along with P values. P < 0.05 was considered significant without adjustment for multiple tests.

A separate analysis was completed to compare the means of immune markers of patients in the acute phase of disease (day post PCR <14) between participants who were later determined to be with or without PASC, dyspnea, fatigue, confusion, difficulty concentrating, forgetfulness, joint pain and chest pain. Independent T-tests were used for this analysis. All analyses were performed with SAS 9.4 (Cary, NC) or stats package in R.

## 3 Results

### 3.1 Participant characteristics and demographic data

The 67 participants included in this analysis had a mean age of 51, and 42 (63%) were female (**Table 1**). The mean body mass index (BMI) was 29.75, categorized as overweight. Those who suffered from PASC had a significantly higher BMI than those without PASC, as discussed in more detail in *Results 3.5.* Those with severe and moderate COVID-19 were significantly more likely to develop PASC than those with mild disease (**Table 1**). Most participants (N=38, 57%) suffered from mild COVID-19, while 16 had moderate disease (24%), and 13 (19%) had severe disease. The most frequently reported PASC symptom was fatigue, followed by forgetfulness or absent-mindedness (**Table 1**).

**Table 1:** Baseline Demographics.

Characteristics	Total	PASC	No PASC	P-value
	N = 67	N=33	N=34	
Age, mean (SD), y	51 ± 17·5			
Sex, no. (%)				
Female	44 (66)	23 (70)	21 (62)	0.214
Male	22 (33)	10 (30)	13 (38)	0.263
BMI, mean (SD)	29·75 ± 7·8	33.13	27.19	0.032
Ethnicity, no. (%)				
Non-Hispanic/Latinx	56 (84)	27 (81.81)	29 (85)	0.48
Hispanic/Latinx	11 (16.41)	6 (18.18)	5 (15)	
Disease Severity*, no. (%)				
Mild	38 (57)	9 (27)	29 (85)	0.04
Moderate	16 (24)	13 (39)	3 (9)	0.003
Severe	13 (19)	11 (33)	2 (6)	0.038
Co-existing conditions, no. (%)				
COPD	2 (3)	1 (3)	1 (3)	0.851
DM	10 (15)	7 (21)	3 (9)	0.051
HTN	16 (24)	9 (27)	7 (21)	0.724
CAD	3 (4)	1 (3)	2 (6)	0.628
Asthma	10 (15)	6 (18)	4 (12)	0.064
Vaccinated, no. (%)	20 (30)	5 (15)	15 (44)	0.829

*Note: Disease severity was determined by oxygen use in accordance with the Yale Impact Score: mild (no oxygen required), moderate (1–5 L oxygen use), severe (>5 L oxygen use). Pre-existing conditions were collected from electronic hospital records and self-reported at clinic visits: COPD, chronic obstructive pulmonary disease; DM, diabetes mellitus; HTN, hypertension, and CAD, coronary artery disease. Values are presented as mean ± standard deviation or frequency (percent). BMI, Body Mass Index; PASC, post-acute sequelae of COVID-19.

### 3.2 T cells and T cell activation status according to post-acute sequelae of COVID-19 symptoms

To investigate the total number of T-cells and the suite of markers for T-cell activation status in participants with PASC, PBMCs were stained to evaluate for T cells and subpopulations, as well as intracellular cytokines including CD4^+^, CD4^+^CD25^+^, CD4^+^HLADR^+^, CD4^+^IFNγ^+^, CD4^+^IL10^+^, CD4^+^TNFα^+^, CD8^+^, CD8^+^CD38^+^, CD8^+^PD1^+^, CD8^+^GranzymeB^+^, CD8^+^IFNγ^+^, CD8^+^IL10^+^, CD8^+^Ki67^+^, CD8^+^TNFα^+^, and CD8^+^IFNγ^+^TNFα^+^ cells.

We did not detect statistically significant differences at all time points, nor did we observe a trend indicating a distinct change from the acute phase to the subacute phase in intracellular cytokine levels across cell surface markers in those with any PASC symptoms compared to those without PASC. PASC symptom type variation and definitions are evolving (8), and the sample size is a limitation for broad spectrum assessment of T cell responses associated with all types of PASC. There was also no association between clustered neurologic symptoms with T cells when compared to those without neurologic symptoms.

Given the wide range of symptoms reported in our cohort and that symptoms may differ in causative pathogenesis, we elected to next evaluate differences between inflammatory markers in those with and without individual symptoms. The CD4 and CD8 T cells were compared in participants with and without symptoms of fatigue, dyspnea, forgetfulness or absent-mindedness, confusion, difficulty concentrating, chest pain, and joint pain. Participants were included in the analysis twice when there were two longitudinal study visits greater than 30 days apart.

**Figure 1:**
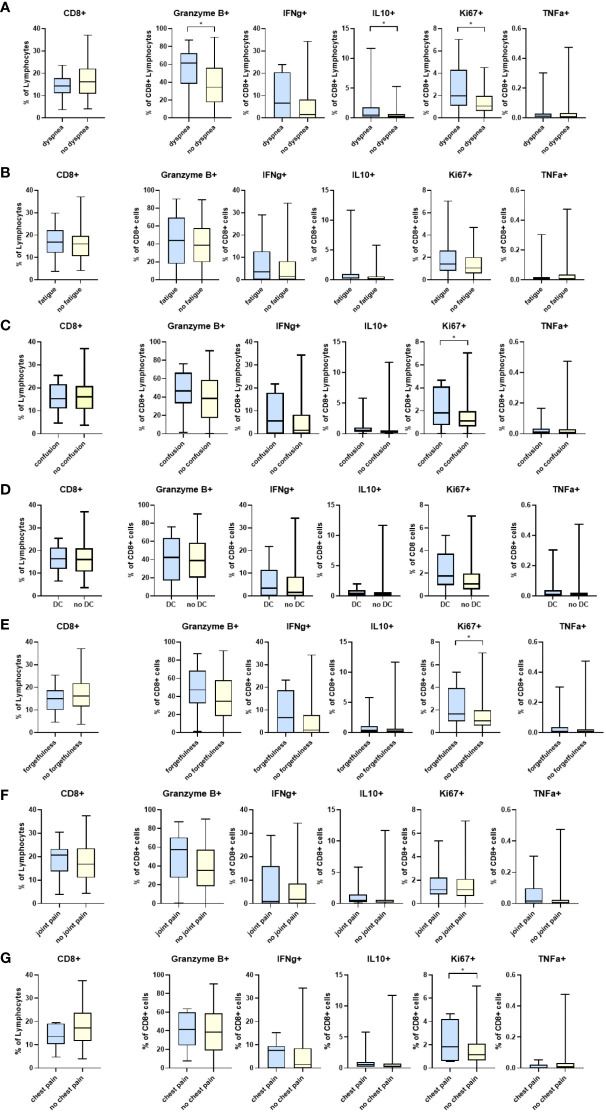
Differences in CD8 T cell markers and inflammatory cytokines in participants reporting symptoms of: **(A)** dyspnea (N=20) and without dyspnea (N=104), **(B)** fatigue (N=35) and without fatigue (N=89), **(C)** confusion (N=17) and without confusion (N=107), **(D)** difficulty concentrating (DC) (N=26) and without difficulty concentrating (N= 98), **(E)** forgetfulness or absent-mindedness (N=28) and without forgetfulness or absent-mindedness (N=96), **(F)** joint pain (N=23) and without joint pain (N=101), and **(G)** chest pain (N=8) and without chest pain (N=116). *p<0.05 DC=Difficulty concentrating.

Participants who reported difficulty breathing had significantly higher levels of CD8^+^CD38^+^ cells than those who did not (25·9 vs. 17·4, p=0·05) (**Supplementary Table 1**). Additionally, Granzyme B^+^, IL10^+^, and Ki67^+^ cytotoxic T lymphocytes (CTLs) were also significantly higher in the group reporting persistent dyspnea (**Figure 1**). Proliferating T cells (CD8^+^Ki67^+^) cells were significantly higher in those reporting dyspnea, forgetfulness, confusion, and chest pain. The percentage of CD4^+^CD25^+^ cells was significantly higher in participants with persistent forgetfulness, chest pain, and joint pain (**Supplementary Figure 1**). There were significantly higher CD4^+^ T cells in participants that had difficulty concentrating, but no differences in CD4^+^IFNγ^+^, IL10^+^, TNFα^+^ or HLADR^+^ when evaluated in those with and without symptoms of PASC (**Supplementary Figure 1**).

### 3.3 Inflammatory markers as predictors of post-acute sequelae of COVID-19 in acutely infected individuals

Of the 67 participants included in this analysis, 24 were enrolled during the acute phase of their illness and continued in the study through the convalescent phase. Most of the participants (N=13, 54·2%) had severe COVID-19 based on the Yale scale for oxygen requirement. Of these 24 participants, 18 (75%) developed PASC and 6 (25%) did not. We evaluated differences in intracellular inflammatory markers and CD4 and CD8 surface markers in the acute phase of illness between cases that developed PASC versus cases that did not develop PASC. We found that CD8^+^Ki67^+^ cells were significantly higher at the time of acute illness in those who went on to develop persistent dyspnea than those who did not (2·84 ± 1·97 vs. 1·25 ± 0·73, p=0·02). CD4^+^CD25^+^ cells were significantly higher in the acute phase of COVID-19, as a percentage of total lymphocytes, in participants who later developed persistent forgetfulness when compared to those who did not (p<0·01). CD4^+^ cells were also significantly higher during the acute phase of illness in those who developed persistent difficulty concentrating (p=0·02).

### 3.4 Longitudinal evaluation of inflammatory markers and post-acute sequelae of COVID-19 symptoms

**Figure 2:**
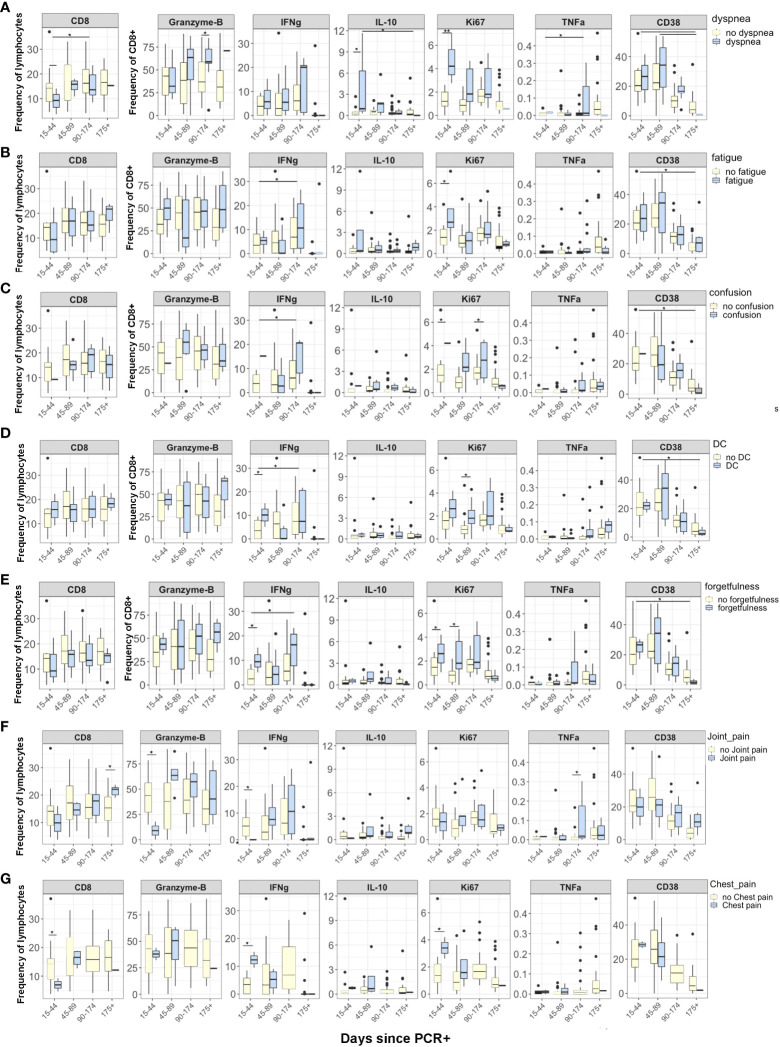
Differences in CD8 T cell markers and inflammatory cytokines in participants grouped according to days from SARS-CoV-2 PCR+ test. Reporting symptoms included dyspnea (15-44, N=3; 45-89, N=5; 96-174, N=5; 175+, N=1) and without dyspnea 15-44, N=11; 45-89, N=18; 96-174, N=24; 175 +, N=16); Fatigue (15-44, N=3; 45-89, N=6; 96-174, N=8; 175+, N=3) and without fatigue (15-44, N=10; 45-89, N=17; 96-174, N=21; 175+, N=14); Confusion (15-44, N=1; 45-89, N=7; 96-174, N=3; 175+, N=1) and without confusion (15-44, N=12; 45-89, N=16; 96-174, N=27; 175+, N=17); Difficulty concentrating (DC) (15-44, N=2; 45-89, N=7; 96-174, N=6; 175+, N=2) and without difficulty concentrating (15-44, N=11; 45-89, N=16; 96-174, N=24; 175+, N=16); and forgetfulness or absent-mindedness (15-44, N=3; 45-89, N=9; 96-174, N=9; 175+, N=2) and without forgetfulness or absent-mindedness (15-44, N=10; 45-89, N=14; 96-174, N=20; 175+, N=16). Statistical significance was calculated using ANOVA with Tukey HSD. P<0.05 was considered significant.

We next sought to evaluate inflammatory markers longitudinally in correlation with persistent symptoms. Participants were categorized into four time points according to the number of days since their positive SARS-CoV-2 PCR test (15-44, 45-89, 90-174, and 175+ days post- SARS-CoV-2 PCR+ test). We compared the CD8^+^ T cell pattern and activation status in participants who were positive or negative for individual PASC symptoms over time. Overall, there was no difference longitudinally in inflammation markers. However, several markers were different between groups at the 15–44-day time point at the 15-44 days post-PCR+, CD8^+^Ki67^+^ cells were significantly higher in participants with dyspnea (4·32 vs. 1·13, p=0·01), fatigue (3·56 vs. 1·09, p = 0·041), confusion (4·12 vs. 0·04, p=0·001), and forgetfulness (3·41 vs. 0·97, p=0·04) than in participants without these symptoms (**Figure 2**). Additionally, we observed significantly higher CD8^+^IL10^+^ cells in patients with dyspnea compared to patients without dyspnea at 15-44 days after PCR+ (1·19 vs. 0·98, p=0·02). IFNγ levels were significantly higher (p=0·05) in patients with any cognitive symptoms (confusion, difficulty concentrating, and forgetfulness) than those without any reported cognitive symptom 15-44 days after PCR+. There were no significant differences in CD4^+^ cell markers or intracellular cytokines longitudinally when examined with specific symptoms (**Supplementary Figure 2**).

Although not examined statistically due to small sample sizes, we plotted the intracellular inflammatory markers across 4 time points for both groups to visualize possible trends in participants with PASC symptoms. We plotted the CD8^+^Ki67^+^, Granzyme-B, and IFNγ levels in each individual patient over time. CD8^+^Ki67^+^ cells were nearly stable in patients without symptoms but were significantly higher in patients with dyspnea, fatigue, confusion, difficulty concentrating, and forgetfulness 15-44 days post-PCR+, stabilized between 45 and 174 days, and then began to decline after 175 days (**Figure 2**). Interestingly, CD8^+^Granzyme-B^+^ cells increased longitudinally in patients with dyspnea and forgetfulness from 15 to 175 days post-PCR+ but did not change over time in patients without persistent symptoms. IFNγ had a distinct pattern compared to Ki67 and Granzyme-B: CD8^+^ IFNγ^+^ cells varied between 15- and 174-days post-PCR but thereafter began to decline in PASC+ patients.

### 3.5 Body mass index associated with post-acute sequelae of COVID-19 and inflammatory markers

Body mass index (BMI) at the time of enrollment was strongly associated with several symptoms of PASC in our cohort, including fatigue (BMI 34·6 vs 27·9, p<0·001), joint pain (34·8 vs 29·2, p=0·03), difficulty breathing (35·8 vs 28·6, p=0·001), forgetful or absent-mindedness (35·1 vs 28·1, p<0·01), confusion (36·8 vs 28·9, p<0·01), and difficulty concentrating (34·7 vs 28·7, p<0·01). A trend was observed between BMI and elevated T cell and inflammatory markers, including CD4^+^CD25^+^, CD8^+^IFNγ^+^, and CD8^+^Granzyme B^+^ cells, but was not statistically significant. Statistical significance was calculated using ANOVA with Tukey HSD.

### 3.6 Longitudinal analysis of anti-receptor binding domain IgG and post-acute sequelae of COVID-19

**Figure 3:**
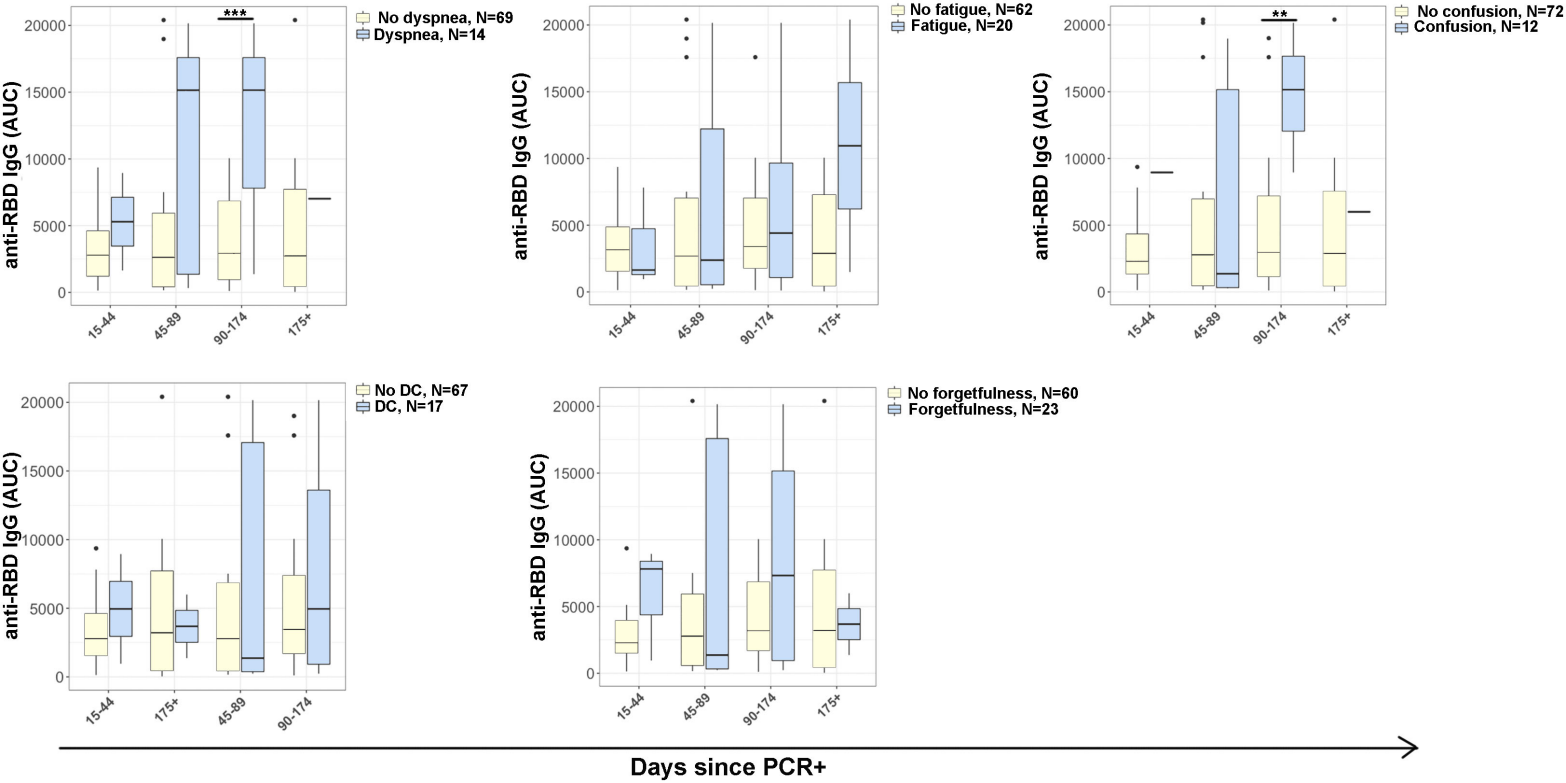
Antibody responses in COVID-19 disease progression. IgG antibody reactivity to SARS-CoV-2 receptor-binding domain (RBD) in plasma samples. Plot representing area under the curve (AUC) was calculated using plasma dilutions 1/250, 1/1250, and 1/6250 of anti-RBD IgG. Reporting symptoms included: dyspnea (15-44, N=3; 45-89, N=5; 96-174, N=5; 175+, N=1) and without dyspnea (15-44, N=11; 45-89, N=18; 96-174, N=24; 175+, N=16); Fatigue (15-44, N=3; 45-89, N=6; 96-174, N=8; 175+, N=3) and without fatigue (15-44, N=10; 45-89, N=17; 96-174, N=21; 175+, N=14); Confusion (15-44, N=1; 45-89, N=7; 96-174, N=3; 175+, N=1) and without confusion (15-44, N=12; 45-89, N=16; 96-174, N=27; 175+, N=17); Difficulty concentrating (DC) (15-44, N=2; 45-89, N=7; 96-174, N=6; 175+, N=2) and without difficulty concentrating (15-44, N=11; 45-89, N=16; 96-174, N=24; 175+, N=16); and e) absent-mindedness (15-44, N=3; 45-89, N=9; 96-174, N=9; 175+, N=2) and without forgetfulness or absent-mindedness (15-44, N=10; 45-89, N=14; 96-174, N=20; 175+, N=16). Statistical significance was calculated using ANOVA with Tukey HSD at each time point. P<0.05 was considered significant. **p<0.01, ***p<0.001.

ELISA was performed for anti-receptor binding domain (RBD) IgG to evaluate correlation with common PASC symptoms (**Figure 3**). ELISAs were completed at each participant visit, and subsequently analyzed by days post positive SARS-CoV-2 PCR. At day 90-174 post PCR, anti-RBD IgG was significantly higher in participants who reported confusion (p<0·012) and dyspnea (p<0·001) than in those who did not report symptoms.

### 3.7 Viral titers

Of the 67 participants included in this study, 24 participants were enrolled during the acute phase of their illness. We obtained nasopharyngeal SARS-CoV-2 PCR data on all patients, and only 13 of the 24 had a high enough cycle threshold to proceed to viral culture by plaque assay. Due to this small number of individuals with viral titer data, there were no significant differences in viral titer levels in those with and without any individual symptom of PASC or inflammatory markers.

## 4 Discussion

Persistently elevated inflammatory T cell markers may contribute to the development and progression of multiple PASC symptoms (33, 34). The CD8^+^Ki67^+^ cells from a longitudinal observational cohort in the Northern Colorado COVID-19 Biorepository were increased for the following individual symptoms of dyspnea, forgetfulness, confusion, and chest pain. Interestingly, CD8^+^Ki67^+^ cells were higher during the acute phase of COVID-19 in those who went on to develop persistent dyspnea and could be used as a predictor of PASC. As a marker of cellular proliferation, an increase in Ki67^+^ CTLs indicates continued CD8^+^ T cell activation and response (35, 36). Other investigators have also demonstrated an association of increased cytotoxic memory T cells with PASC (37). Additionally, IL10^+^ CTLs were increased in those with symptoms of dyspnea, consistent with chronic infection as a down regulator of inflammation. Similarly, CD4^+^CD25^+^ cells represent an increase in T regulatory cells in those with long-term symptoms of forgetfulness, chest pain, and joint pain, and suggest an ongoing attempt to control host inflammation. CD4^+^CD25^+^ cells are also more elevated in the acute phase of COVID-19 in those who later develop persistent symptoms and could be used as a predictor of PASC. However, studies investigating additional characteristics of the CD4^+^CD25^+^ cells are essential to differentiate activated T cells from regulatory T cells.

We found that increases in intracellular cytokines were most striking in participants who complained of persistent dyspnea. Severe acute respiratory syndrome coronavirus 2 infects alveolar macrophages which in turn activate T cells in the alveolar space, which could in part explain why patients with dyspnea may have more inflammation than those without (38). Other studies have shown that activated SARS-CoV-2 specific CD8+ T cells are associated with decreased lung function (33). Together these findings strongly suggest that dyspnea is closely related to persistent inflammation.

Alternatively, persistent inflammation in patients with PASC could be due to continued or intermittent viral antigen exposure or be part of an autoimmune response. Patients with PASC have been found to have detectable SARS-CoV-2 antigen in plasma (39), which could lead to continued immune activation. Viruses such as chikungunya, Ebola virus (EBOV), Zika virus, measles virus, and others may persist in viral sanctuary sites such as the reproductive tract, CNS, and synovial tissue (40–44). Severe acute respiratory syndrome coronavirus 2 could remain in the host in a similar manner, causing intermittent viremia and persistent inflammation. An incomplete host immune response may lead to diminished viral clearance. We did not have many participants with low PCR cycle threshold values or culturable virus during convalescent follow up visits, however these samples were only obtained in the nasopharynx, and though virus may be rapidly cleared there, it does not preclude virus presence in alternative sites.

Decreased levels of nucleocapsid peptide-specific CD8^+^CD107a^+^ T cells are also associated with PASC (29), which could be suggestive of poor immune response and viral clearance. Alternatively, proliferating CTLs could be a part of an autoimmune process (45). An increase in autoimmune antibodies has been observed in those with PASC (46, 47). Though we were not able to obtain nasopharyngeal samples early enough in the disease process to detect culturable virus in most patients, others have found that high SARS-CoV-2 viral loads at the time of COVID-19 diagnosis are predictors of PASC (47), and persistent inflammation could also be associated with virus-induced immune dysregulation which takes many months to resolve. Our findings showing increased levels of Ki67 in CTLs as a predictor of PASC support this theory.

Interestingly, investigators have sought to determine if genetic loci are associated with elevations of T cell markers in patients with severe COVID-19 disease through single-cell RNA-sequencing and transcriptomics (48–50). These same methods could be applied to those with persistent inflammation in PASC to determine if similar genetic loci are present and could assist with predicting those who may go on to develop PASC.

The COVID-19 survivors reporting persistent symptoms following acute infection and determined to have PASC had increased CD4^+^ and CD8^+^ T cell activation for up to 180 days post SARS-CoV-2 infection. Participants with ongoing dyspnea had the most notable change in CD8^+^ T cell markers, further suggesting that symptoms of shortness of breath are most likely related to chronic inflammation. Additionally, we found that participants who developed PASC had higher levels of CD8^+^Ki67^+^ cells during acute illness, and this marker could be investigated as a predictor of PASC.

Chronic inflammation is a risk factor for other chronic conditions, such as cardiovascular, cerebrovascular, or autoimmune disease, which heightens the significance of follow up investigation with this cohort (51, 52). The data from this study support a growing body of evidence associating PASC with persistent inflammation and immune dysregulation as early predictors of disease and warrant attention towards appropriate therapeutics and interventions for the prevention and treatment of PASC.

Limitations of the study: The sample size was small for this immune profiling analysis, with only 67 participants included, and our study attrition involved 13 patients that did not follow up for subsequent visits after the first convalescent visit. Another limitation to consider was the variation in vaccination status through the study period. Some participants were vaccinated towards the end of our study (from December 30, 2020, to April 5, 2021), and inflammation associated with immune response to immunization could influence the interpretation of results. Notably, there were minimal differences in inflammatory markers between those with and without PASC, when all symptom types were combined, which prompted the further investigation of T cell responses by individual symptoms that are included in the broad definition of PASC. *Post hoc* analysis could be considered as a limitation to this study; however, corrections for multiple comparisons were part of the statistical analysis. This study has utility for statistical power calculations needed to assess T cell signatures in future human studies investigating inflammation following respiratory viral infections that are associated with PASC development.

## Data availability statement

The raw data supporting the conclusions of this article will be made available by the authors, without undue reservation.

## Ethics statement

The studies involving humans were approved by CSU’s Research Integrity and Compliance Review Board (IRB protocol ID 2105 and 20-10063H), and the UCH IRB (Colorado Multiple IRB 20–6043). The studies were conducted in accordance with the local legislation and institutional requirements. The participants provided their written informed consent to participate in this study. Ethical approval was not required for the studies on animals in accordance with the local legislation and institutional requirements because only commercially available established cell lines were used.

## Author contributions

SML: Conceptualization, Data curation, Formal Analysis, Investigation, Methodology, Validation, Writing – original draft, Writing – review & editing. TSD: Conceptualization, Data curation, Formal Analysis, Investigation, Methodology, Writing – original draft, Writing – review & editing. KM: Formal Analysis, Validation, Writing – review & editing. BAB: Data curation, Investigation, Project administration, Writing – review & editing. TLW: Conceptualization, Data curation, Investigation, Writing – review & editing. KB: Data curation, Investigation, Writing – review & editing. SS: Data curation, Investigation, Writing – review & editing. BMS: Conceptualization, Formal Analysis, Writing – review & editing. JD: Conceptualization, Formal Analysis, Funding acquisition, Investigation, Project administration, Supervision, Writing – review & editing. MHT: Conceptualization, Data curation, Funding acquisition, Investigation, Methodology, Supervision, Validation, Visualization, Writing – review & editing. EPR: Conceptualization, Data curation, Formal Analysis, Funding acquisition, Methodology, Project administration, Supervision, Validation, Visualization, Writing – review & editing. MT: Data curation, Project administration, Writing-review & editing.

## Supplementary material

Supplementary Table 1 Immune markers with significant differences in participants with and without PASC symptoms. FOP, frequency of the parent population; FOL, frequency of the total lymphocyte population (FOL).

Supplementary Figure 1 Differences in CD4 T cell markers and inflammatory cytokines in participants reporting the following symptoms: **(A)** dyspnea (N=20) and without dyspnea (N=104), **(B)** fatigue (N=35) and without fatigue (N=89), **(C)** confusion (N=17) and without confusion (N=107), **(D)** difficulty concentrating (DC) (N=26) and without difficulty concentrating (N= 98), **(E)** forgetfulness or absent-mindedness (N=28) and without forgetfulness or absent-mindedness (N=96).

Supplementary Figure 2 Differences in CD4 T cell markers and inflammatory cytokines in participants grouped based on days from SARS-CoV-2 PCR+ test: **(A)** dyspnea (N=20) and without dyspnea (N=104), **(B)** fatigue (N=35) and without fatigue (N=89), **(C)** confusion (N=17) and without confusion (N=107), **(D)** difficulty concentrating (DC) (N=26) and without difficulty concentrating (N= 98), **(E)** forgetfulness or absent-mindedness (N=28) and without forgetfulness or absent-mindedness (N=96). Statistical significance was calculated using ANOVA with Tukey HSD. P<0.05 was considered significant.
