# Supplementary material for: Persistent CD8+ T cell proliferation and activation in COVID-19 adult survivors with post-acute sequelae: a longitudinal, observational cohort study of persistent symptoms and T cell markers
Source: Front Immunol. 2024 Jan 23;14:1303971. doi: 10.3389/fimmu.2023.1303971 (PMC10848319; doi:10.3389/fimmu.2023.1303971)
Supplement: Supplementary Table 1 — Immune markers with significant differences in participants with and without PASC symptoms. FOP, frequency of the parent population; FOL, frequency of the total lymphocyte population (FOL). [file DataSheet_1.pdf]

Supplementary Table 1. Immune markers with significant differences in participants with and without PASC symptoms. Note: FOP=frequency of the parent population; FOL=frequency of the total lymphocyte population (FOL)

**Immune Markers with and without Symptoms**			
**T cell marker or cytokine**	**Symptom present** (**%**±SD)	**Symptom not present** (**%**±SD)	**P-value**
	Difficulty breathing	*No difficulty breathing*	
$CD8^+CD38^+$ ‡	25·9 ± 16·6	17·4 ± 13·6	0·05
$CD8^+GranzymeB^+$ ‡	55·0 ± 25·8	38·0 ± 23·1	0·02
$CD8^+IL10^+$ ‡	1·9 ± 3·2	0·4 ± 0·5	0·002
$CD8^+Ki67^+$ ‡	2·5 ± 2·0	1·2 ± 0·8	0·0004
	Confusion	*No confusion*	
$CD8^+Ki67^+$ ‡	2·3 ± 1·6	1·3 ± 1·2	0·02
	Difficulty concentrating	*No difficulty concentrating*	
$CD4^+$†	32·1 ± 8·7	26·5 ± 9·7	0·04
	Forgetfulness	*No forgetfulness*	
$CD4^+CD25^+$ †	0·3 ± 0·2	0·2 ± 0·1	0·02
$CD8^+Ki67^+$ ‡	2·0 ± 1·4	1·2 ± 1·2	0·02
	Chest pain	*No chest pain*	
$CD4^+CD25^+$ ‡	1·2 ± 1·2	0·7 ± 0·6	0·05
$CD8^+Ki67^+$ ‡	2·5 ± 1·6	1·3 ± 1·2	0·03
	Joint pain	*No joint pain*	
$CD4^+CD25^+$ †	1·3 ± 1·2	0·6 ± 0·5	0·0009

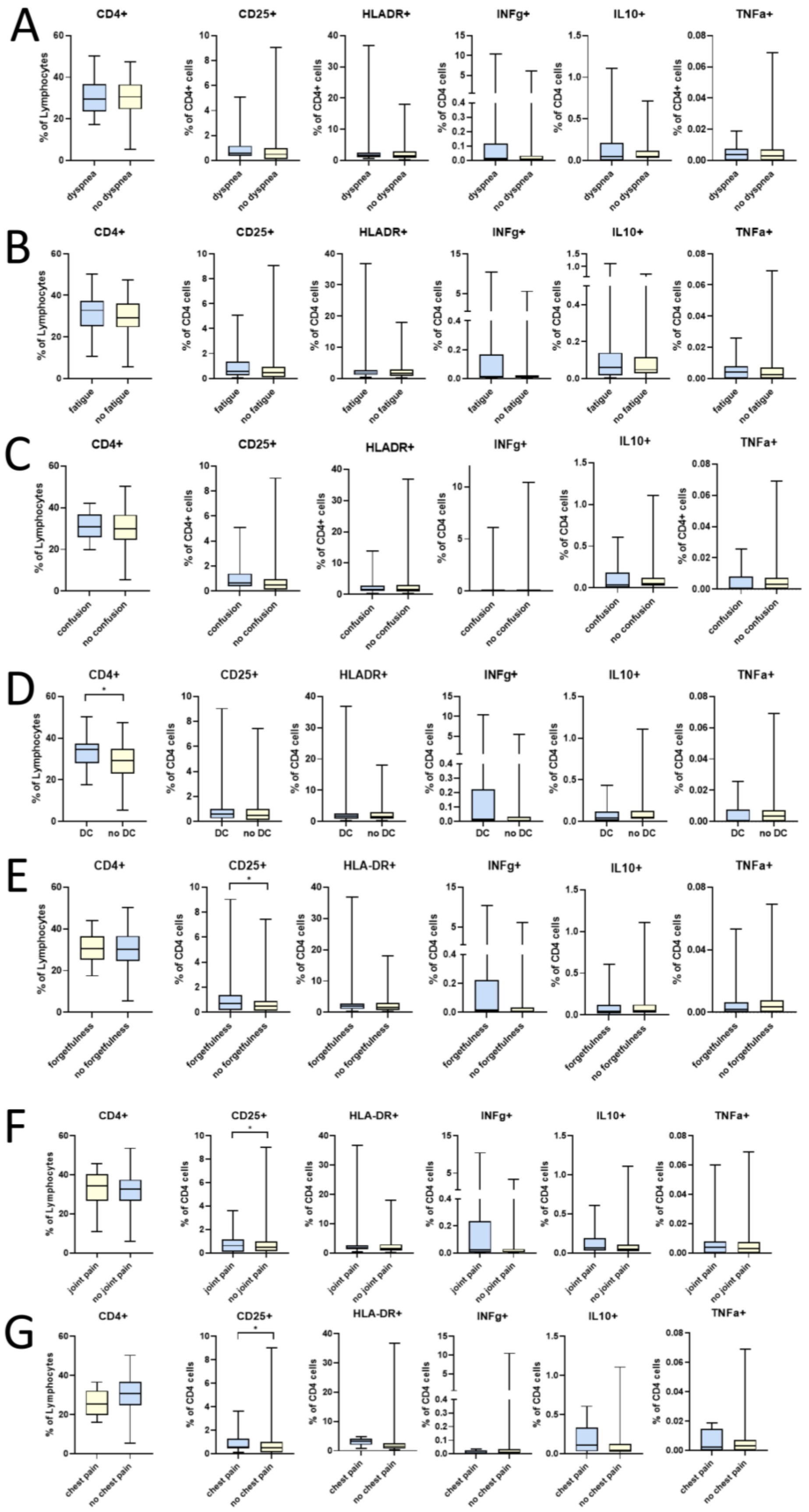

Supplementary Figure-1: Differences in CD4 T cell markers and inflammatory cytokines in participants reporting the following symptoms: (A) dyspnea (N=20) and without dyspnea (N=104), (B) fatigue (N=35) and without fatigue (N=89), (C) confusion (N=17) and without confusion (N=107), (D) difficulty concentrating (DC) (N=26) and without difficulty concentrating (N= 98), (E) forgetfulness or absentmindedness

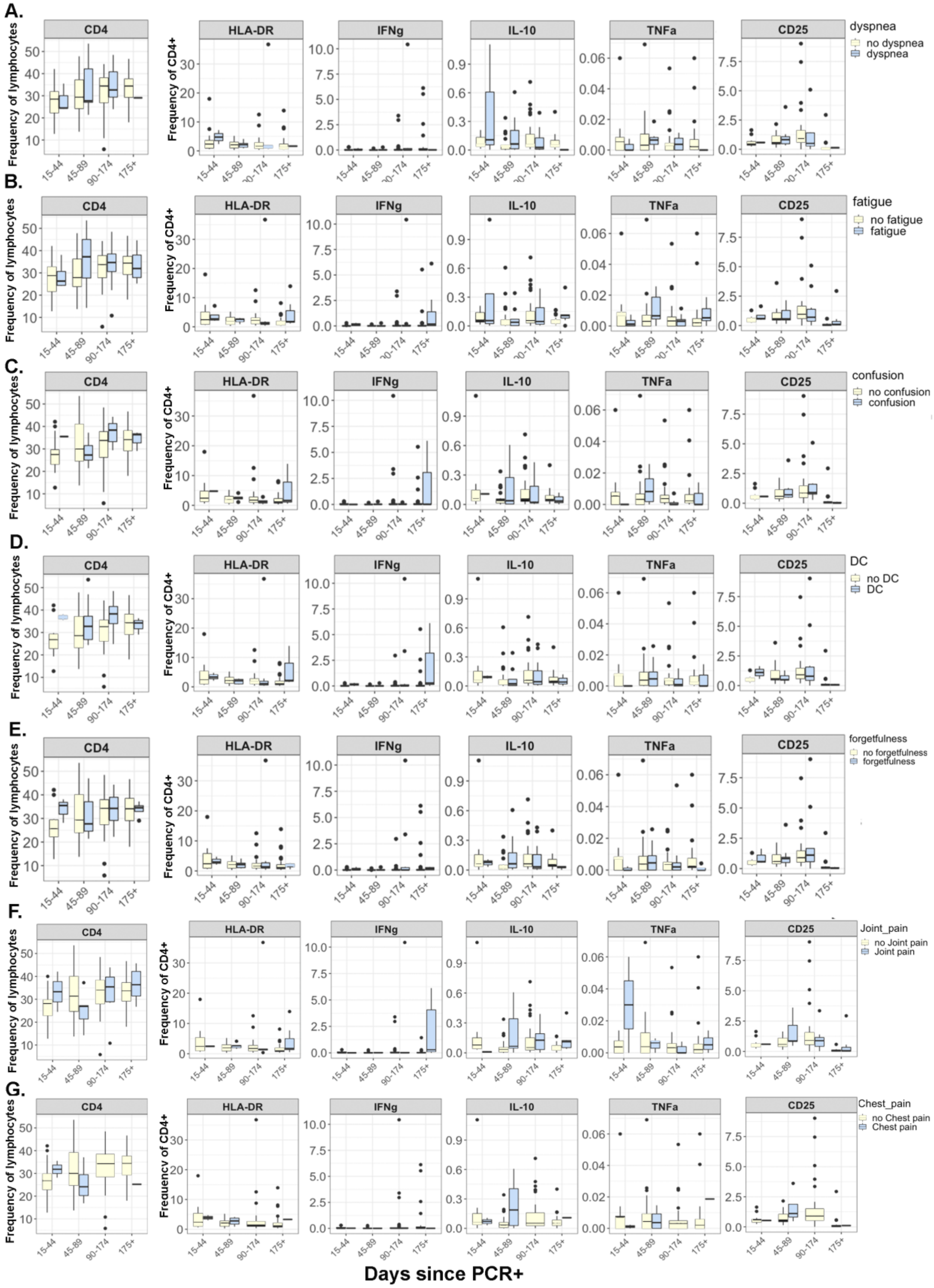

Supplementary Figure 2: Differences in CD4 T cell markers and inflammatory cytokines in participants grouped based on days from SARS-CoV-2 PCR+ test: (A) dyspnea (N=20) and without dyspnea (N=104), (B) fatigue (N=35) and without fatigue (N=89), (C) confusion (N=17) and without confusion (N=107), (D) difficulty concentrating (DC) (N=26) and without difficulty concentrating (N= 98), (E) forgetfulness or absent-mindedness (N=28) and without forgetfulness or absent-mindedness (N=96). Statistical significance was calculated using ANOVA with Tukey HSD. P<0.05 was considered significant.
